# A new genus and species in the tribe Empoascini (Hemiptera, Cicadellidae, Typhlocybinae) from China

**DOI:** 10.3897/zookeys.386.7020

**Published:** 2014-03-07

**Authors:** Si-han Lu, Dao-zheng Qin

**Affiliations:** 1 Key Laboratory of Plant Protection Resources and Pest Management of the Ministry of Education, Entomological Museum, Northwest A&F University, Yangling, Shaanxi 712100, China

**Keywords:** Homoptera, Auchenorrhyncha, Cicadelloidea, new taxa, taxonomy

## Abstract

One new leafhopper genus, *Circinans*, is described with a new species *Circinans striata* **sp. n.** as the type species from southern China. Habitus photos and illustrations of male genitalia of this new species are given and differences between the new genus and closely related genera are discussed.

## Introduction

The tribe Empoascini is a diverse group and differs from other leafhoppers in the subfamily Typhlocybinae in lacking an appendix in the forewing and in having a submarginal vein at the apex of the hindwing and veins RP, MP’ confluent distally (Dietrich 2005). Currently, Empoascini comprises more than 1,000 described species, widely distributed in every continent except Antarctica. In agroecosystems, some species in the tribe, and especially *Empoasca* spp., may attack a broad range of host plants and induce “hopper burn” in the plant tissue. *Empoasca* leafhoppers may also vector viruses, bacteria, and fungi and transmit them efficiently to plants as a consequence of their ingestion–egestion feeding behavior (Backus et al. 2005).

The fauna of Empoascini in China is very rich. Recent studies of this tribe include Lu et al. (2013), Qin et al. (2013) and Yu and Yang (2013a, 2013b) with more than 180 Chinese species in 32 genera having been reported so far. However, the Chinese fauna of this tribe remains obscure and many new taxa await being described. This paper adds a new genus and species based on our recent examination of materials collected from southern China.

## Material and methods

The specimens examined in this study are deposited in the Entomological Museum, Northwest A & F University, Yangling, Shaanxi, China (NWAFU). The entire male abdomens of the examined specimens were removed and cleared in 10% NaOH and drawn based on preparations preserved in glycerin. Figures of male genitalia were drawn using an OLYMPUS PM-10AD, and wings were drawn using a LEICA MZ-12.5 microscope. External morphology was observed under an OLYMPUS SZX-10 microscope. Photographs of the specimens were made using a Nikon SMZ 1500 microscope with a Retiga 4000R camera (CCD). Images were produced using the software Auto-Montage Pro. All pictures were edited using Adobe Photoshop CS7.0 (Adobe Systems). Body measurements are from apex of vertex to tip of forewing.

Morphological terminology follows Zhang (1990) with the following exceptions: wing venation follows Dworakowska (1993), terms for the four types of setae on the subgenital plate and “horn” for the posterior extension of the dorsum of pygofer follow Southern (1982).

## Taxonomy

### Circinans

Qin & Lu gen. n.

http://zoobank.org/9F7211A6-3786-4013-A45D-C779BD48B7F4

http://species-id.net/wiki/Circinans

#### Type species.

*Circinans striata* Qin & Lu, sp. n. here designated.

#### Description.

**Figures 1–8.**
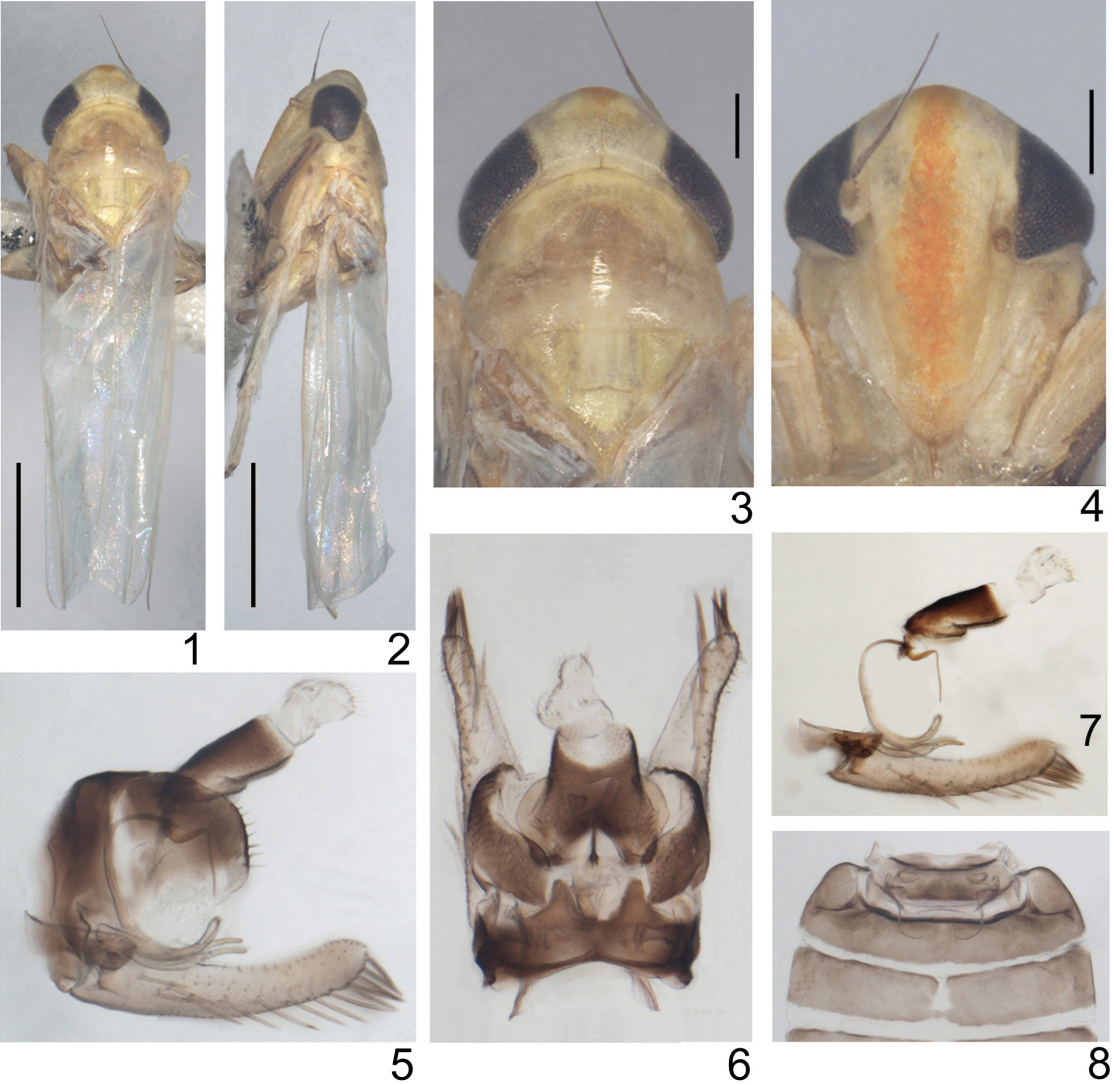
*Circinans striata* Qin & Lu, sp. n. **1** male adult (abdomen removed), dorsal view **2** same, left lateral view **3** head and thorax, dorsal view **4** face **5** male genitalia, left lateral view **6** same, dorsal view **7** anal tube and anal styli, aedeagus, connective, parameres and subgenital plates, left lateral view **8** abdominal apodeme. Scale bars = 1 mm (Figs 1, 2); 0.2 mm (Figs 3, 4).

**Figures 9–18.**
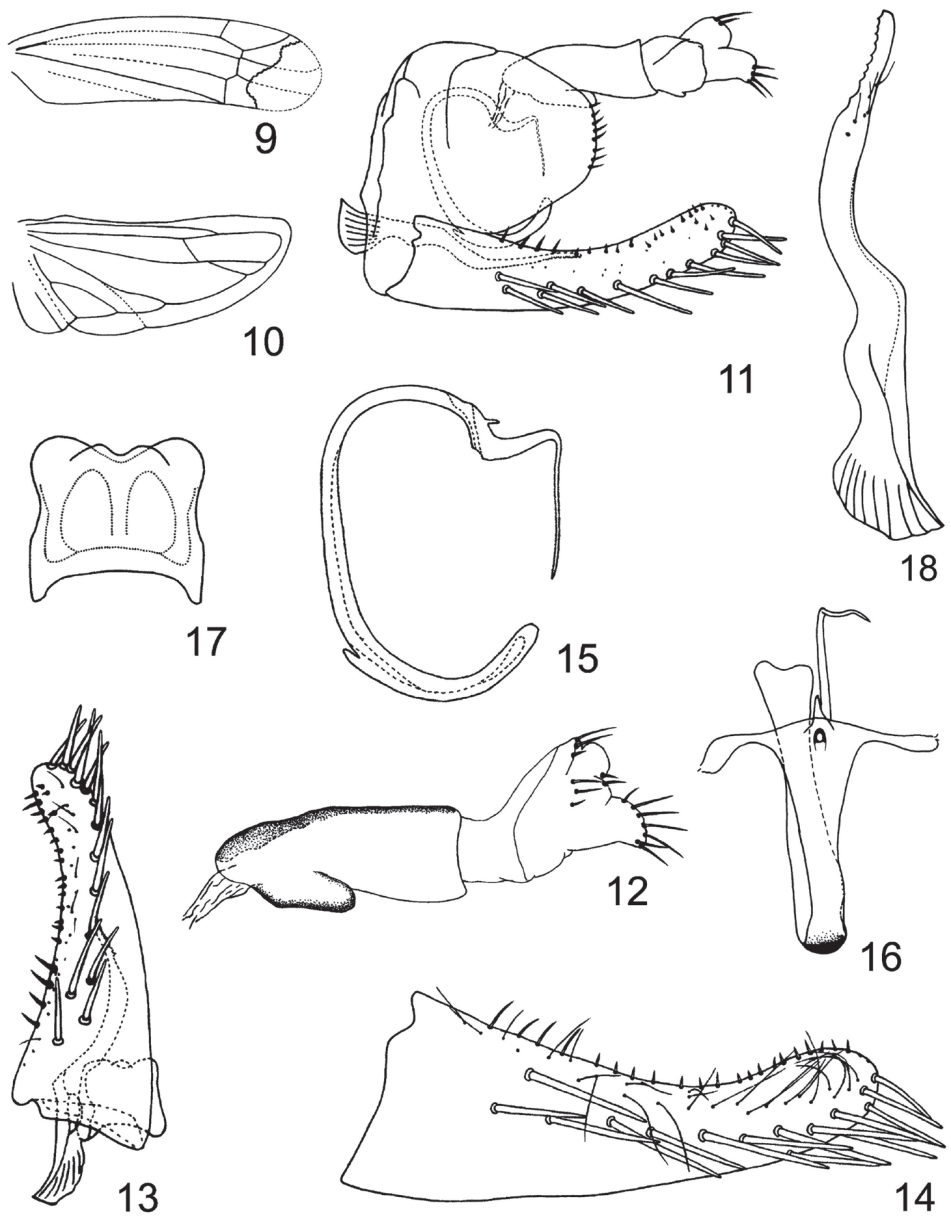
*Circinans striata* Qin & Lu, sp. n. **9** forewing **10** hindwing **11** male genitalia, left lateral view **12** anal tube and anal styli, left lateral view **13** paramere, connective and subgenital plate, dorsal view **14** subgenital plate, dorsal view **15** aedeagus, left lateral view **16** aedeagus, dorsal view **17** connective **18** paramere.

Small, yellowish empoascines. Head including eyes as wide as pronotum (Figs 1, 3). Vertex in midline shorter than width between eyes, anterior margin slightly produced medially (Figs 1, 3), profile of transition of vertex to face rounded, coronal suture distinct, extending well beyond crown midlength (Figs 2, 3). Face broad, convex in profile, lateral frontal suture distinct (Figs 2, 4). Ocelli on margin about equidistant between eye and midline (Figs 3, 4). Pronotum large (Figs 1, 3). Forewing narrow, rounded apically, apical cells occupying nearly one-third total length, veins RP and MP’ dissociated at their bases, both arise from r cell and MP’’+CuA’ from m cell, c and r cells nearly equal in width, both narrower than m and cua cells (Fig. 9). Hindwing with CuA branched, point of branching distad of coalescence of CuA with MP” (Fig. 10).

Male basal abdominal sternal apodemes not well developed (Fig. 8). Male pygofer short, with few rigid microsetae along posterior margin, ventral appendage absent (Figs 5, 11), dorsal bridge short (Fig. 6). Subgenital plate far exceeding pygofer side, widest at base and tapered to rounded apex, all categories of setae present, A-group setae near base of plate, B-group setae occupying more than half length of anterior margin, C-group setae bluntly terminated, arranged in two rows near base and subapically, between them the macrosetae merged into a single row (Figs 5, 7, 11, 13, 14). Paramere robust, slightly longer than pygofer, apophysis bearing prominent dentifer and long setae situated more cephalad (Figs 5, 11, 18). Connective lamellate (Figs 13, 17). Aedeagus in profile narrow, C-shaped in outline, preatrium well developed (Figs 5, 7, 11, 15, 16). Anal tube process broad and extended caudad (Figs 5, 7, 11, 12).

#### Etymology.

This name alludes to the unique shape of the aedeagus. Gender: feminine.

#### Discussion.

In the Empoascini, *Circinans* is similar to *Alebrasca* Hayashi & Okada, *Luodianasca* Qin & Zhang, *Membranacea* Qin & Zhang, *Nikkotettix* Matsumura, *Schizandrasca* Anufriev and *Szara* Dworakowska in having all apical cells in forewing with separate bases, branching point of CuA in hindwing at or distad of coalescence of CuA with MP” and male pygofer lacking ventral appendage (in *Nikkotettix*, 3rd apical cell in forewing stalked or sessile, male pygofer having or lacking ventral appendage). However, the new genus differs from these genera in having the C-shaped aedeagus; C-group setae of subgenital plate arranged in two rows subapically (aedeagus not C-shaped and C-group setae of subgenital plate uniseriate subapically in these other genera), from *Luodianasca* and *Szara* in the subgenital plate having A-group setae (A-group setae absent in *Luodianasca* and *Szara*). This new genus also differs from *Alebrasca* and *Membranacea* in the presence of an anal tube appendage (anal tube appendage absent in *Alebrasca* and *Membranacea*).

#### Distribution.

China (Fujian, Guizhou).

### Circinans striata

Qin & Lu sp. n.

http://zoobank.org/CB84160F-C396-4DC7-B4BA-DBA0CA9CAA52

http://species-id.net/wiki/Circinans_striata

Figs 1 –18

#### Description.

Size. Male 3.7–4.1 mm.

General color of body brownish yellow. Vertex yellow, with large creamy patches surrounding ocelli caudomesad, coronal suture brown. Eye black. Face with a longitudinal orange stripe in middle which is less distinct at apex of anteclypeus, rest area of face yellow except genae whitish. Antennae with scape yellow, pedicel brownish. Pronotum brownish yellow centrally and whitish posteriorly, surrounded by irregular arc of light hypodermal pattern in addition to three irregular creamy patches along anterior margin. Centre of scutellum with a quadrate whitish patch anteriorly, a triangular creamy patch caudad of scutoscutellar sulcus and two triangular beige patches at basal angles on each side. Forewing and hindwing subhyaline. Dorsum of abdomen blackish, ventrally sordid yellow. Legs yellow except 1st and 2nd tibia and tarsus brown.

Basal sternal abdominal apodemes parallel sided, not reaching end of segment 3 (Fig. 8). Male pygofer in lateral view with rounded apex, bearing 7–9 rigid setae along posterior margin (Figs 5, 11); dorsum of pygofer deeply emarginated caudo-medially, dorsal bridge nearly quarter of the total length of pygofer, horns broad, widely separate basally and diverging slightly (Fig. 6). Subgenital plate in lateral aspect with apical 2/5 curved dorsad, A-group setae (4) longer and slightly thicker than B-group, B-group setae (19–20) occupying almost 2/3 length of anterior margin, relatively evenly distributed to apex of plate, C-group setae (14–15) starting at 1/4 distance from base and extending to apex of plate, D-group setae (22–25) roughly biseriate, starting basodorsad of subgenital plate (Figs 5, 7, 11, 13, 14). Paramere narrowed in apical half, dentifer bearing 9–10 prominent teeth and about 4 long setae and few sensory pits on lateral surface near apex (Fig. 18). Preatrium of aedeagus fairly long in lateral aspect, arched subbasally and subapically, subapical protrusion of aedeagus tapering and afterwards strongly bent ventrad, terminal part of aedeagus needle-shaped, slightly sinuate, gonopore subterminal, ventral, almost at the same level the aedeagus with a small process on the dorsal side (Figs 5, 7, 11, 15, 16). Connective quadrate, broader than long, anterior margin notched medially, lateral margins slightly concave submedially (Figs 13, 17). Anal tube process in lateral view short, originating near basoventral angle of segment X and extending caudad, gradually narrowing with rounded apex (Figs 5, 7, 11, 12).

**Female.** Unknown.

#### Type materials.

**Holotype.** ♂ (NWAFU), China, Fujian Province, Wuyi Mountain, 16 August 2008, coll. X. Gao and X. T. Li, by light trap. **Paratype.** 1♂ (NWAFU), China, Guizhou Province, Fanjing Mountain, 28 July 2001, coll. Q. Sun, by light trap.

#### Etymology.

The specific epithet refers to the longitudinal orange stripe on the face.

#### Distribution.

China (Fujian, Guizhou).

## Supplementary Material

XML Treatment for Circinans

XML Treatment for Circinans striata
